# Pollen assemblages in *Tamarix* cone sediments and their implications for environmental change in the Southeastern Qaidam basin over the past 600 years

**DOI:** 10.3389/fpls.2025.1696049

**Published:** 2025-11-24

**Authors:** Xinyu Hou, Yuanjie Zhao, Xiaoqian Guo, Shaoteng Song, Yang Feng, Yaqing Dong

**Affiliations:** 1 School of Geographical Sciences, Hebei Normal University, Shijiazhuang, Hebei, China; 2 Hebei Key Laboratory of Environmental Change and Ecological Construction, Shijiazhuang, Hebei, China; 3 Hebei Technology Innovation Center for Remote Sensing Identification of Environmental Change, Shijiazhuang, Hebei, China

**Keywords:** *Tamarix* cone, pollen, environmental change, quantitative paleoclimate reconstruction, principal component analysis, Qaidam basin

## Abstract

**Introduction:**

The Qaidam Basin is situated in the transitional zone between Kunlun Mountains and Qilian Mountains as well as in China’s low-to-mid-latitude monsoon transition zone. The absence of conventional climatic information carriers has posed significant challenges for environmental change studies in this region. The pollen assemblage in *Tamarix* cone sediments serves as an effective proxy carrier for reconstructing the climosequence in arid areas.

**Methods:**

This study employed ^14^C and ^210^Pb dating, pollen assemblages from a sampling site with pollen samples of *Tamarix* cone sediments, and principal component analysis (PCA) and quantitative paleoclimate reconstruction to reconstruct climatic and environmental changes in the Nuomuhong area from 1444 to 2022 AD.

**Results:**

The pollen is mainly comprised of herbs and shrubs, with a desert shrub vegetation type dominated by *Artemisia*, Chenopodiaceae, *Nitraria*, and Poaceae. Environmental changes are strongly correlated with soil salinity, the temperature of the warmest month (MTWA), the mean annual temperature (TANN) and the temperature of the coldest month (MTCO). Additionally, the changing trend of soil salinity was reconstructed based on the content of *Nitraria*. The following four zones are identified: 1) 1444–1520 AD, had high salinity and was humid (MTWA of 18.6–21.4 °C, TANN of 5.2–7.1 °C, and MTCO of -10.4–-8.9 °C); 2) 1520–1617 AD, with lower salinity and a humid conditions (MTWA of 16.0–19.4 °C, TANN of 3.1–5.8 °C, MTCO of -11.3–-9.6 °C); 3) 1617–1821 AD, with progressive aridification with reduced salinity (MTWA of 16.5–17.4 °C, TANN of 3.5–4.1 °C, MTCO of -11.5–-10.9 °C); and 4) 1821–2022 AD, with increasing salinity and a drier conditions (MTWA of 17.7–19.2 °C, TANN of 4.1–5.5 °C, MTCO of -11.3–-9.8 °C).

**Discussion:**

The environmental changes may be associated with climate and groundwater variations induced by the Little Ice Age. Our findings provide implications for climate reconstruction and studies on environmental change in the Qaidam Basin and other arid regions with strong interannual variability.

## 1 Introduction

Environmental changes under the influence of climate change and human activities are important challenges that human beings need to face at present (Guo et al., 2008). Climate change, such as drought, have affected the characteristics of aeolian deposits and the history of vegetation, which is significant for the study of earth system patterns and the changes of human production and livelihoods (Dupont-Nivet et al., 2007; Herb et al., 2015).

Research on environmental change patterns often relies on meteorological data (Tan et al., 2014), however, meteorological records alone are insufficient to fully reconstruct environmental conditions beyond the past 60 years (Xu, 2017). To overcome this limitation, it is important to select suitable proxy indicators. At present, the common information carriers used to indicate climate are ice cores, lake sediments, marine sediments, stalagmites and tree rings (Chen et al., 2019). By studying Antarctic ice cores, Brook and Buizert (2018) reported that the increase in primary greenhouse gas concentrations has impacted global warming since the Industrial Revolution. Using lake sediments, Chen et al. (2016) reconstructed the precipitation sequences in the Northeastern Tibetan Plateau during the Holocene, showing a trend of first increasing and then decreasing. However, due to the dry climate, scarce water bodies and poor vegetation development, it is difficult to obtain the above information carriers in arid areas, and dating materials are also relatively scarce, which has brought certain difficulties to studying climate change in arid areas (Wang and Zhao, 2011).

The Qaidam Basin is located in the transitional zone between the Kunlun Mountains and the Qilian Mountains in China, and it is an area that is impacted by both the Westerlies and the Asian monsoon, and is a typical arid area. In addition, the Qaidam Basin exhibits high sensitivity to changes in terrestrial ecosystems (Liu et al., 2016; Li et al., 2025). Meteorological observations indicate that since 1961, the basin has experienced a mean annual temperature increase at a rate of 0.53°C per decade (Wang et al., 2014), accompanied by a rise in precipitation and cryospheric retreat (Zhang et al., 2023). These characteristics make it one of the regions on the Qinghai-Tibet Plateau most strongly affected by climate change (Kuang and Jiao, 2016).These changes have led to changes of vegetation in this basin (Zeng and Yang, 2008), and it is an ideal area for investigating pollen and environmental changes (Zhao et al., 2020).

*Tamarix* cones are widely distributed in the arid desert region, the surrounding ancient dry riverbeds, and the low-lying areas; the sedimentary veins of these cones are formed by alternating aeolian sand layers and litter layers, which have good continuity and provide rich proxy information on regional climate and environmental changes (Li et al., 2023). As an important proxy index of paleoclimate reconstruction, pollen assemblages reflect the response relationship between vegetation types and climate (Ge et al., 2017). The *Tamarix* cone sedimentary vines are effective information carriers containing pollen assemblages and dating materials to rebuild the paleoclimate in arid areas, with characteristics of high resolution, environmental sensitivity and multi-index compatibility, providing a unique perspective for the study of environmental change in arid areas.

Therefore, this study focuses on the pollen assemblages in sediments from a *Tamarix* cone in the southeastern Qaidam Basin, employing principal component analysis (PCA) and quantitative paleoclimate reconstruction to investigate the patterns of environmental change and their driving factors in this region. Our objectives are: (1) to establish the age series of the *Tamarix* cone; (2) to analyze the patterns of environmental change and influencing factors in the Qaidam Basin based on variations in pollen assemblages; (3) to discuss the implications of our findings for future research on *Tamarix* cones and their application in studies of environmental change in arid regions.

## 2 Materials and methods

### 2.1 Natural survey of the study area

The sampling site is in the Nuomuhong area southeast of the Qaidam Basin, and it has a continental climate, with dry, windy and cold characteristics. The sampling site has a mean annual temperature (TANN) of approximately 3.5 °C. At present, the annual precipitation (PANN) in this basin is approximately 50 mm/a (Herb et al., 2015; Chen and Bowler, 1986), and the average annual wind speed is greater than 3.7 m/s (Wang et al., 2006). There is plenty of sunshine and strong evaporation around the sampling site, the average annual evaporation rate is 1353.9 - 3526.1mm/a. The evaporation rate exceeds the replenishment from precipitation, making this area usually in a state of drought (Bibi et al., 2019). The Gobi Desert is the main landform. The vegetation coverage in the Qaidam Basin generally shows a semi-circular decreasing trend from southeast to northwest inland. The average vegetation coverage is 12.9%, which is relatively low and dominated mainly by *Tamarix ramosissima*, *Alhagi* sp*arsifolia* and *Phragmites australis* (Zhang, 2019).

### 2.2 Sample collection

**Figure 1:**
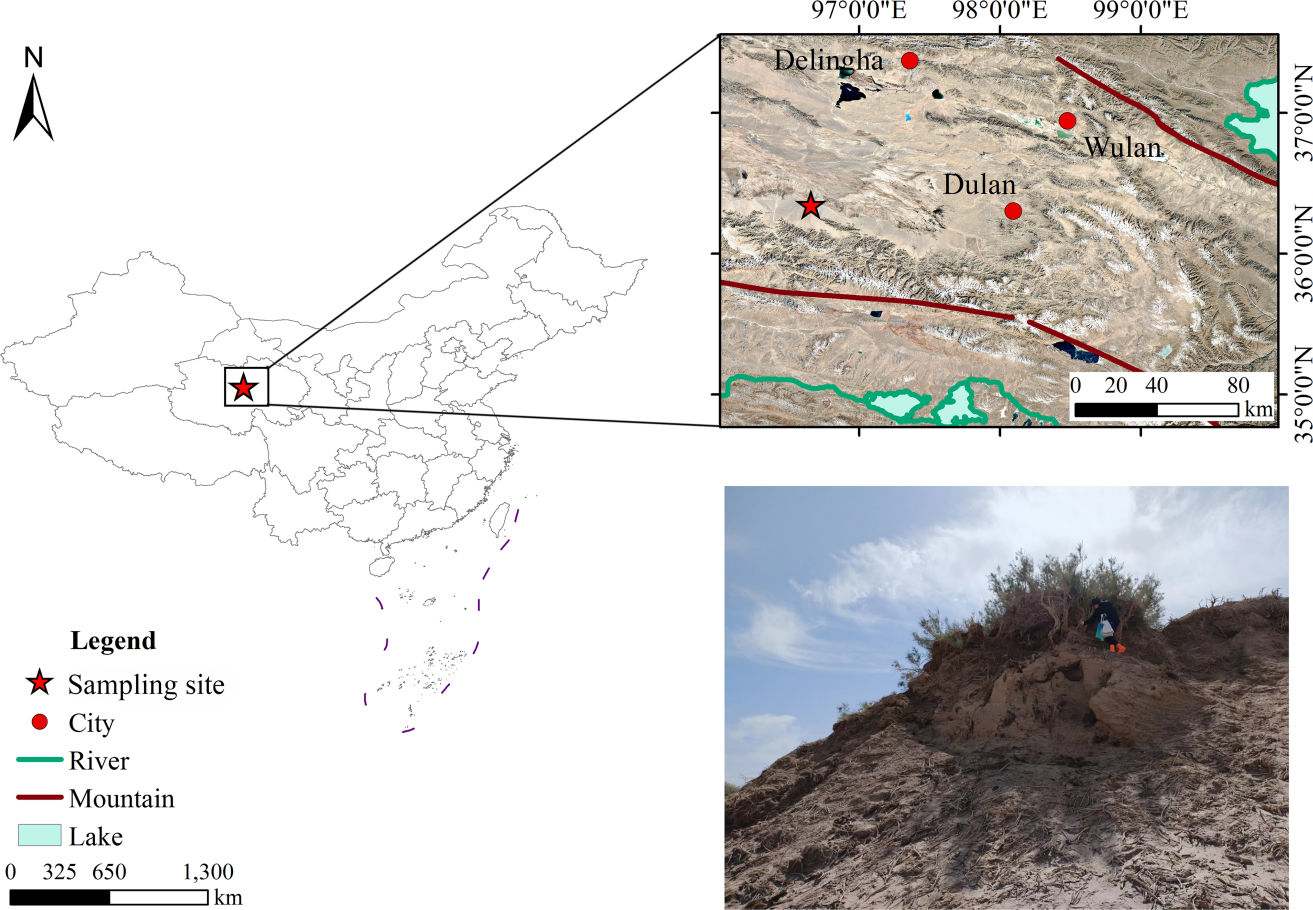
Sketch map of the study area and profile of *Tamarix* cone sediments.

In the Nuomuhong area (36˚ 20′ 55.5248″ N, 96˚ 39′ 26.6368″ E; at an altitude of 2783 m; **Figure 1**), *Tamarix* cone sediments are continuously distributed, *Tamarix* cone sediments are continuously distributed, with the heights of these sediments ranging from tens of centimeters to tens of meters. From September 20 to October 2, 2023, a *Tamarix* cone with good growth at the top and shrub height of approximately 1–2 m was selected for sampling. When sampling, the loose sediments on the surface, with no obvious lithologic change and a lower degree of chemical weathering of the *Tamarix* cone were removed until an obvious sediment profile was exposed. After trimming the profile, stratified sampling was conducted from top to bottom, with an average thickness of approximately 4 cm. The depth of the profile was 182 cm. A total of 26 dating samples and 48 pollen samples were collected.

### 2.3 Dating of sediments

Dating using ^210^Pb was completed in the Key Laboratory of Sedimentation and Environment, Nanjing Institute of Geography and Limnology, Chinese Academy of Sciences. In order to verify the results of ^210^Pb dating, we selected 2 leaf samples from the litter layer of the *Tamarix* cone at the lower part of the profile at 176 cm and 182cm for ^14^C dating, which was conducted by Beta Analytic (USA). The δ^13^C values of the leaf samples are -25.1‰ and -26‰, respectively, we used the IntCal20 calibration dataset for calendar year correction of the leaf samples. Based on the ^210^Pb and ^14^C dating results, we constructed the age depth model of *Tamarix* cone at the Nuomuhong sampling site. The test results are shown in **Table 1**.

**Table 1:** Results of ^210^Pb and ^14^C dating of *Tamarix* cone sediments in the Nuonuhong area.

Sample No.	Depth (cm)	^210^Pbex specific activity(Bq/kg)	Sample No.	Material	δ^13^C (‰)		Depth (cm)	^14^C Age (cal yr B.P.)
N1	1	49.79	N176	Plant	-25.1		176	466 ± 30
N2	6	39.34	N182	Plant	-26.0		182	489 ± 30
N3	13	34.06						
N4	20	35.30						
N5	26	39.99						
N6	33	32.06						
N7	40	20.69						
N8	47	30.95						
N9	54	20.09						
N10	62	9.62						
N11	70	18.21						
N12	77	17.79						
N13	85	26.10						
N14	93	18.00						
N15	100	19.31						
N16	107	14.59						
N17	114	10.10						
N18	122	31.54						
N19	129	18.38						
N20	140	16.80						
N21	148	1.96						
N22	155	7.04						
N23	162	3.17						
N24	170	7.73						
N25	176	0.50						
N26	182	1.96						

### 2.4 Pollen identification

The samples (200 g) for pollen analysis were prepared via standard procedures, including removal of siliceous materials with HF and heavy-liquid flotation (Fægri et al., 1989). Exotic *Lycopodium* spores were added (27,560 grains/tablet) as a marker for calculating pollen concentration. The pollen was identified using a Zeiss optical microscope at 400× magnification. For every sample, the sum of pollen exceeded 400 grains. The Pollen Flora of China (Second edition) (Wang et al., 1995) and Illustrated Handbook of Quaternary Pollen in China (Tang et al., 2016) were used as references for pollen identification. Tilia software was used to construct pollen concentration diagrams, and zonation was conducted using CONISS (Grimm, 1987).

### 2.5 Quantitative paleoclimate reconstruction

**Figure 2:**
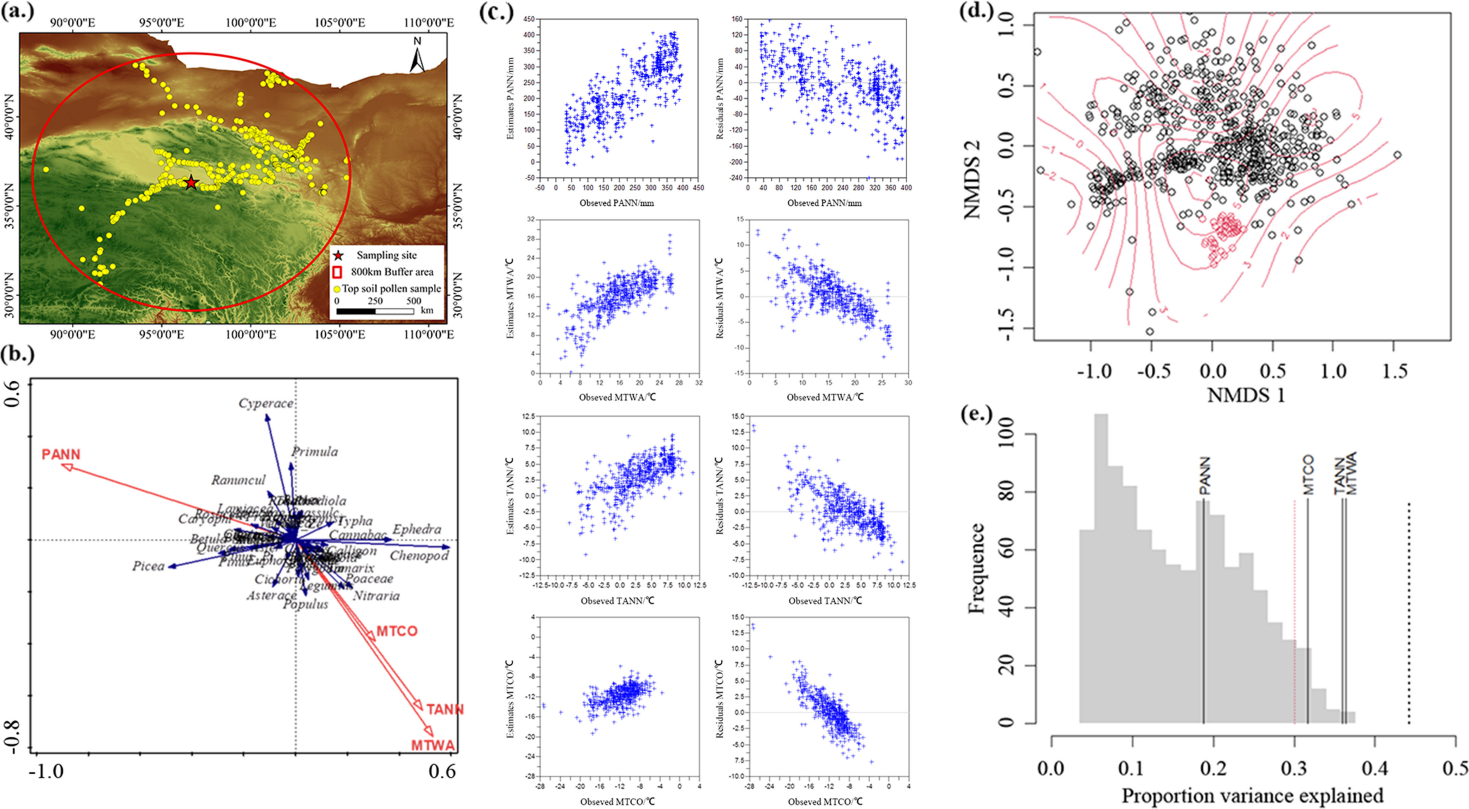
Results of climate reconstruction. **(a)** Locations of surface pollen samples. **(b)** RDA results for the pollen taxa and significant environmental variables controlling the surface pollen compositions. **(c)** Results obtained using the Weighted Averaging Partial Least Squares (WA-PLS) method, with significance test results based on 999 iterations. **(d)** Observed values and residuals between observed and estimated values using WA-PLS. **(e)** Results of significance test for quantitative reconstruction of WA-PLS method based on randomized data.

As the growth patterns of plants are indicative of regional climate, plant pollen serves as a reliable indicator for the quantitative reconstruction of paleoclimate (Ghosh et al., 2015). The transfer function method can effectively reconstruct past environmental and climatic changes with a relatively small number of training data sets (Ghosh et al., 2017). Even when employing regionally restricted pollen-climate calibration sets, this method enables reliable site-specific reconstructions (Birks et al., 2000; Lotter et al., 2000; Herzschuh et al., 2010), making it one of the most commonly used approaches for quantitative paleoclimatic reconstruction during the Holocene. Therefore, we adopt the transfer function method to establish a quantitative, pollen-based climate reconstruction model in the Nuomuhong area. This aims to facilitate the reconstruction of climatic conditions in the southeastern Qaidam Basin and provide a baseline for future studies in other arid regions. To construct a pollen-based climofunction, we used surface pollen samples of the Chinese Topsoil Database, within the ranges of 800 km from the sampling site (Zheng et al., 2008), and excluded 116 samples, with no intention to focus on extreme sites (Ghosh et al., 2017), and finally 586 surface pollen samples have been used as the training set (**Figure 2a**). The climatic data corresponding to the surface pollen samples were obtained from the China Meteorological Science Data Sharing Service (http://data.cma.cn/).

Pollen quantitative reconstruction relies on the identification of key environmental factors that are associated with various surface pollen assemblages. Therefore, we used Canoco 5 software to conduct detrended correspondence analysis (DCA) of the surface soil pollen data and climatic data, selecting either Canonical Correspondence Analysis (CCA) for normally distributed data, or Redundancy Analysis (RDA), according to the gradient length. For gradient lengths <3, RDA is used; for lengths of 3–4, both methods can be used; and for lengths >4, CCA is used. The DCA ordination in this study indicated a gradient length of 2.93, and thus RDA was selected for the ordination analysis. The Weighted Averaging Partial Least Squares (WA-PLS) is the most commonly used method for quantitative paleoclimatic reconstruction during the Holocene. This method effectively accounts for the unimodal response of pollen and utilizes residuals to mitigate bias while enhancing overall performance (Ghosh et al., 2017; Birks, 1998). This method is considered more accurate and reliable in northern China (You et al., 2025). Therefore, we used RStudio software and C2 software to conduct quantitative paleoclimate reconstruction through the WA-PLS method.

## 3 Results

### 3.1 Chronology of *Tamarix* cone sediments

Based on the comprehensive analysis of ^210^Pb and ^14^C dating data, the age sequence of *Tamarix* cone sediments was established. Because the sampling time occurred in September 2023 AD, the top vein of the *Tamarix* cone sediments was set as 2022 AD, considering the lag of sediment formation. According to the ^210^Pbex specific activity, the average sedimentation rate and age of the samples were calculated using (Equations 1–3) as follows:

(1) $$lnC=-\lambda D/R+lnC_{I}$$

(2) $$S_{A}=S\times (-\lambda )$$

(3) $$\text{T}=T_{\text{A}}-D/R$$

where C is the concentration of ^210^Pb in each sample; D is the depth of each sample; R is the deposition rate of sediment; C_I_ is the initial concentration of ^210^Pb; S_A_ is the average deposition rate of the *Tamarix* cone sediments; S is the slope; T is the age of each sample; and T_A_ is the age of initial concentration.

**Figure 3:**
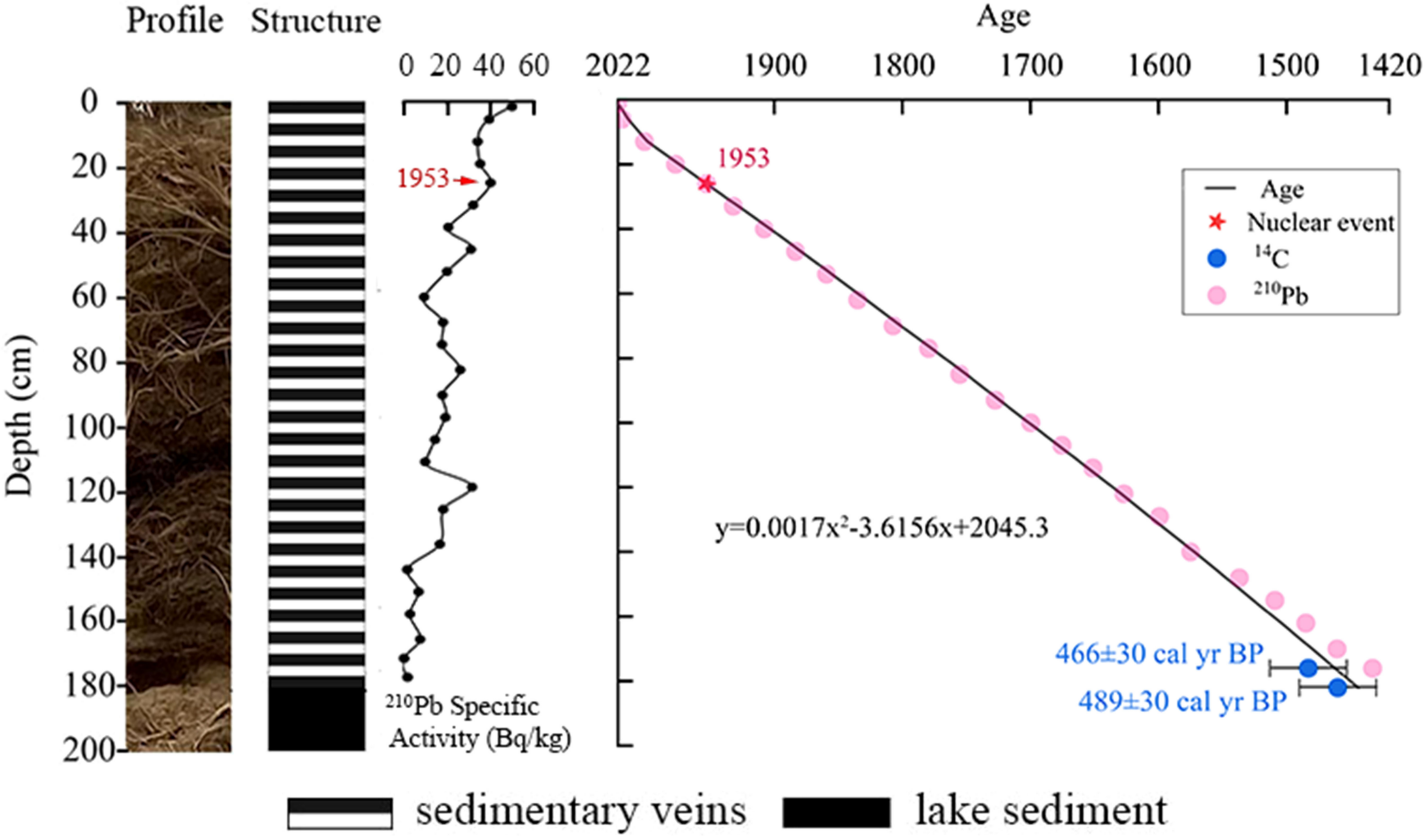
Age sequence of *Tamarix* cone sediments.

Combining the results of ^14^C dating and the time when the first hydrogen bomb test in the Soviet Union produced the peak value of ^210^Pb specific activity in 1953, a linear regression model of sediment depth and age was established. The age series of *Tamarix* cone sediments at the Nuomuhong sampling site was determined to be 1444 AD (**Figure 3**).

### 3.2 Pollen assemblages

**Figure 4:**
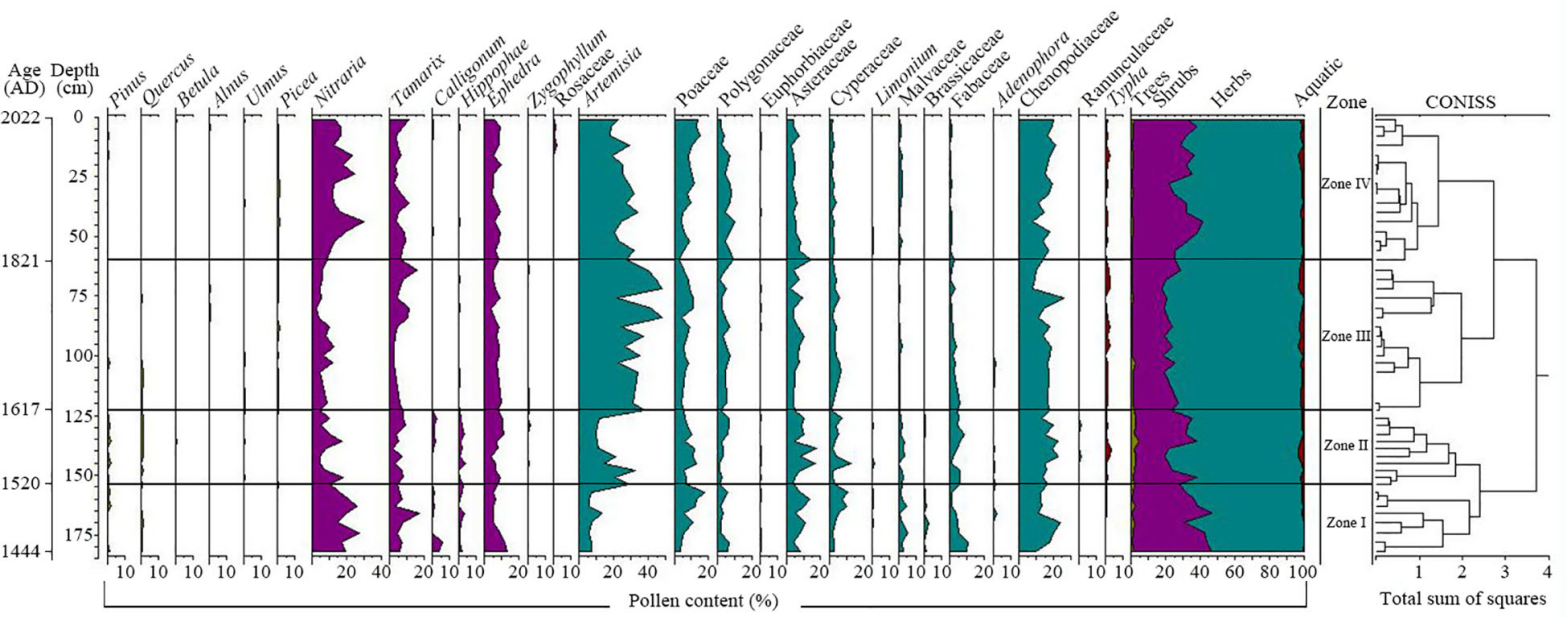
Pollen percentage diagram for the Nuomuhong area.

**Figure 5:**
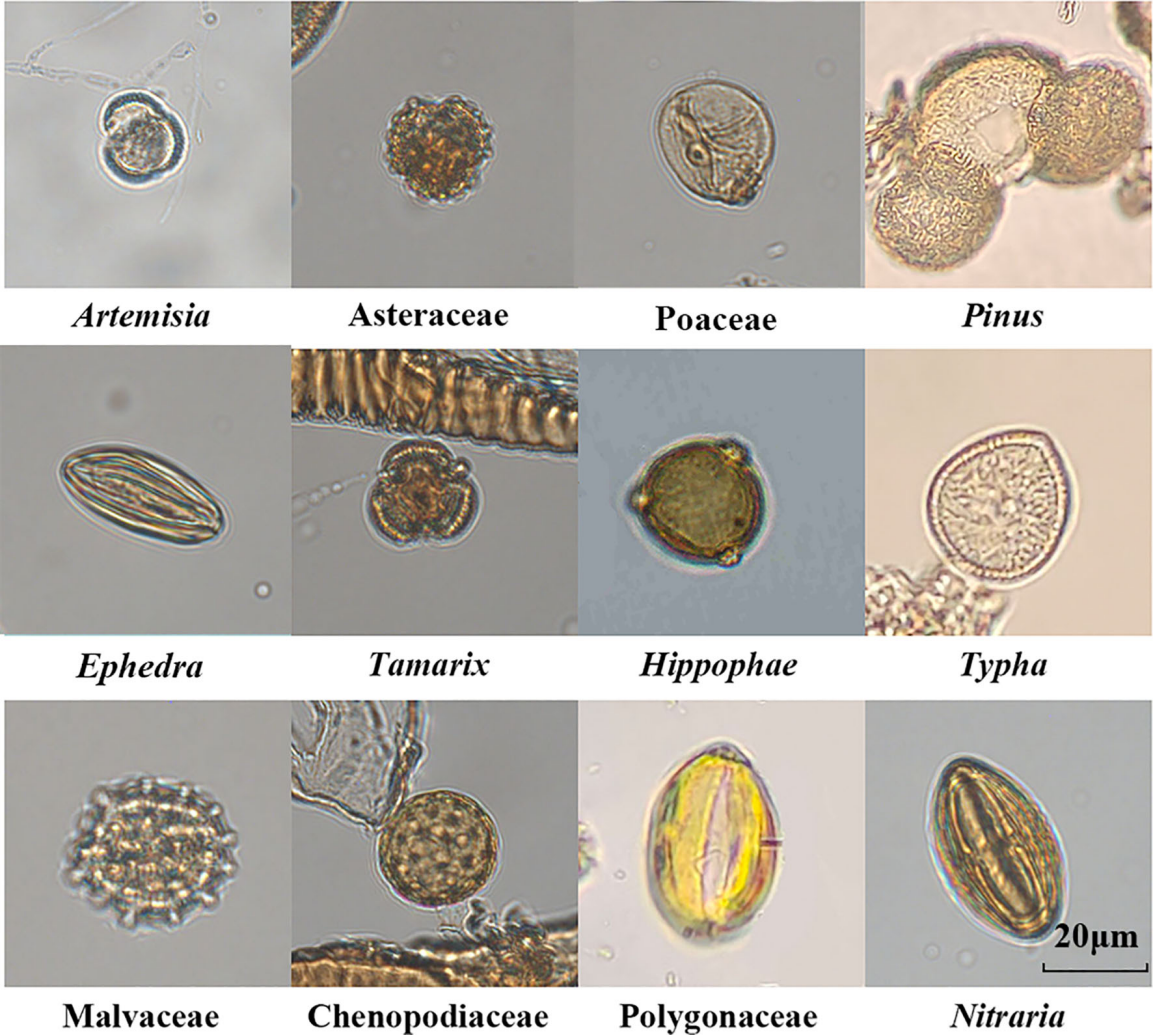
Microscope photos of main pollen species morphology of the Nuomuhong area.

In total, 19,200 pollens were identified from 48 samples, and they were grouped into 27 families or genera as follows: 6 tree genera; 7 shrub families or genera; 14 herbaceous families or genera; and 1 aquatic plant genus (**Figure 4**). Among the main pollen groups in the *Tamarix* cone sediments in the Nuomuhong area, the proportion of herbaceous plants was the highest (average of 68.97%), including mainly *Artemisia* (23.55%; 5.5–48%), Chenopodiaceae (16.14%; 7.75–26.25%), Poaceae (8.43%; 2.75–18%), Asteraceae (7.2%; 2–17.75%), Polygonaceae (4.86%; 1.5–10.25%) Cyperaceae (3.75%; 1.25–12.5%), Fabaceae (2.65%; 0–10.75%), and Malvaceae (1.7%; 0–5.5%). The shrubs were ranked second, with an average of 28.51%, and they included mainly *Nitraria* (12.96%; 2.5–30.25%), *Ephedra* (7.57%; 3.75–13.5%), *Tamarix* (6.46%; 2–17.25%), and *Hippophae* (0.79%; 0–4.25%). The average value of trees was 1.32%, including mainly *Pinus* (0.51%; 0–2.5%). The proportion of aquatic plants was the least (average of 1.21%), including mainly *Typha* (1.21%; 0–3.25%). The microscope photos of main pollen species morphology are shown in **Figure 5**.

Four pollen assemblage zones were identified. Zone I (182–157 cm, 1444–1520 AD) contained 8 samples. Of the four zones, Zone I exhibited the lowest pollen concentration (average of 584 grains/g). Pollen assemblage analysis revealed that herb pollen types were predominant, with an average content of 59.69% (52.25–69.00%). In Zone I, the main herbaceous taxa was Chenopodiaceae (average of 15.09%), followed by Poaceae (9.34%), and *Artemisia* (7.34%) accounted for a lower proportion than that in other zones. Shrub pollens accounted for the highest proportion (29.00–46.25%) in the four zones, with an average content of 38.53%. The main taxa were *Nitraria* (20.38%) and *Tamarix* (7.50%). The average pollen content of trees was 1.41%, and the pollen content of aquatic plants was the lowest (0.38%).

Zone II (157–126 cm, 1520–1617 AD) had 10 samples. In Zone 2, the pollen concentration was higher than that in Zone I, with an average of 1462 grains/g. Herb pollen was predominant, with an average content of 69.35% (60.25–77.75%). The pollen content of *Artemisia* (average of 17.13%) increased to a percentage similar to the highest content of Chenopodiaceae (17.63%). The pollen content of shrubs decreased, with an average content of 26.85% (16.75–36.25%). The main shrub taxa were *Nitraria* (9.88%), *Ephedra* (8.25%) and *Tamarix* (5.83%), but the contents of *Nitraria* and *Tamarix* were lower than those in Zone I. The contents of trees (2.35%) and aquatic plants (1.45%) also increased.

Zone III (126–64 cm, 1617–1821 AD) contained 14 samples. The pollen concentration continued to increase to 2157 grains/g, which was the highest among the four zones. Herb pollen was predominant (average of 76.25%; 69.25–81.00%), and the *Artemisia* pollen content continued to increase to 35.30% (21.75–48.00%). In addition, the Chenopodiaceae pollen content was 15.48% (7.75–26.25%). The average content of shrub pollen was 21.21% (17.50–27.75%), and the contents of *Ephedra* (7.27%), *Nitraria* (6.95%) and *Tamarix* (6.32%) increased. The average content of aquatic plants increased to the highest of the four zones (1.61%). However, the average content of tree pollen decreased to the lowest of the four zones (0.93%).

Zone IV (64–0 cm, 1821–2022 AD) had 16 samples. The pollen concentration decreased to 1717 grains/g, and the herb pollen decreased to 67.00% (57.25–76.75%). Moreover, the *Artemisia* pollen decreased to 25.38%, and the Chenopodiaceae pollen content remained relatively stable (16.30%). The shrub content increased to 30.91% (21.25–40.50%), which was due mainly to the increase of *Nitraria* (16.45%), but the high content of *Ephedra* (7.34%) and *Tamarix* (6.45%) showed no obvious changes. The contents of aquatic plants (1.13%) and tree pollen (0.97%) did not change compared with those of Zone III.

### 3.3 Principal component analysis of pollens

**Figure 6:**
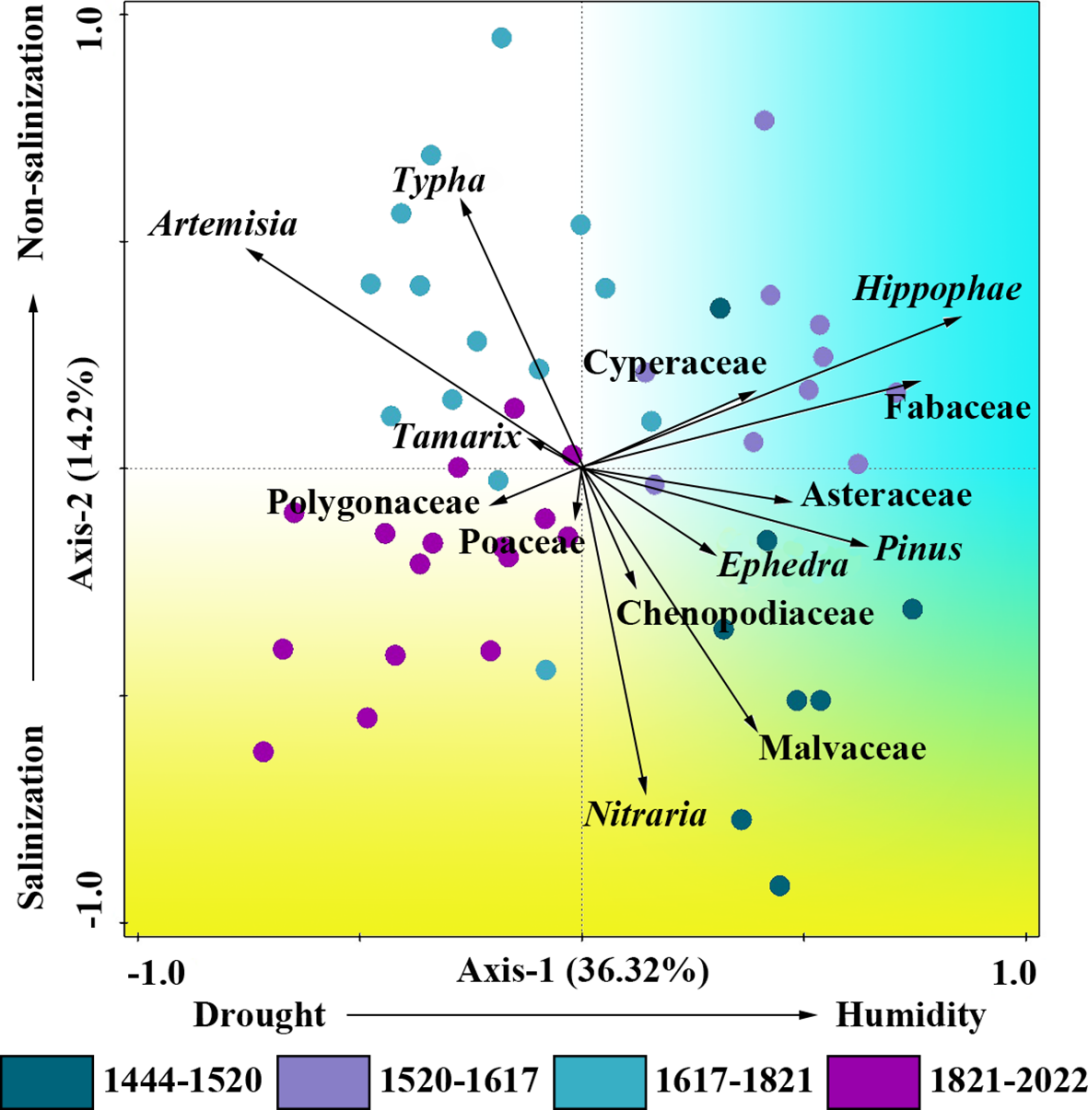
PCA results of the pollen data.

To better reveal the ecological significance of pollen assemblage in the study period, PCA was performed on 14 pollen types with high average contents (more than 0.5%) in 48 pollen samples of the profile (**Figure 6**). The first axis explained 36.32% of the variability, and the first and second axes jointly explained 50.52% of the variability. Among them, the positive direction of the first principal component axis was *Pinus*, Cyperaceae and *Hippophae*, and the negative direction of the first principal component axis was *Tamarix* and *Artemisia*, representing drought-tolerant pollens. Therefore, the first principal component axis mainly reflects changes in humidity, with the positive direction indicating humidity and the negative direction indicating drought. For the second principal component axis, the positive direction was *Nitraria*, and the negative direction was *Typha*. Thus, the second principal component axis mainly reflects changes in salinity, with the positive direction indicating salinization and the negative direction indicating non-salinization.

**Figure 6** illustrates that the points representing 1444 to 1520 AD are mostly positively correlated with the first axis and negatively correlated with the second axis, indicating that the environment during 1444 to 1520 AD was mostly humid and high soil salinity. Most of the points representing 1520 to 1617 AD are positively correlated with the first axis and the second axis, indicating that the environment was relatively humid and the soil salinity was low during 1520 to 1617 AD. Most of the points representing 1617 to 1821 AD are negatively correlated with the first axis and positively correlated with the second axis, indicating that the environment from 1617 to 1821 AD was mostly dry and lower soil salinity. Most of the points representing the period from 1821 to 2022 AD are negatively correlated with the first axis and the second axis, indicating that the environment was dry and high soil salinity during this period.

### 3.4 Quantitative paleoclimatic reconstruction

#### 3.4.1 Results of the screening of climatic factors

The RDA results (**Figure 2b**) indicate that the eigenvalues of the first and second ordination axes are 0.154 and 0.031, respectively, with species-environment correlations of 0.703 and 0.490, respectively. The first and second ordination axes account for 77.98% and 15.59% of the variation in pollen types, respectively (**Table 2**). In the RDA ordination diagram, blue arrows represent species variables, while red arrows represent environmental variables; the direction angle with respect to the ordination axes and the length perpendicular to the vertical direction indicate the degree of correlation between environmental factors and the ordination axes. Notably, PANN is negatively aligned with the first axis, indicating a strong positive correlation with the direction of the first axis, which represents variations in precipitation. The positive direction suggests a drought climate, while the negative direction indicates humidity climate. Pollen types on the first axis are significantly influenced by the moisture levels of the climate, for example, the positive direction predominantly including *Ephedra* and Chenopodiaceae, indicating a relatively drought climate. And the negative direction indicating a relatively dry climate, includes *Picea*. The temperature of the warmest month (MTWA), TANN and the temperature of the coldest month (MTCO) are negatively aligned with the second axis, which represents variations in temperature. The positive direction suggests a cold climate, while the negative direction indicates warm climate. Pollen types on the second axis are influenced by the temperature levels of the climate, with the positive direction including Cyperaceae, indicating a relatively cold climate. And the negative direction includes *Populus* and *Nitraria*, indicating a relatively warm climate. Therefore, PANN, MTWA, TANN and MTCO are key climatic environmental variables controlling the variation in the vegetation types represented by this area.

**Table 2:** Summary statistics of the RDA for pollen types with climatic variables.

	Axis 1	Axis 2	Axis 3	Axis 4	Climatic variables as sole predictor	
					Explained variance (%)	P-value
Eigenvalues	0.1544	0.0309	0.0095	0.0032		
Explained variation (cumulative)	15.44	18.53	19.48	19.81		
Species-environment correlations	0.703	0.4903	0.3206	0.2189		
Variation in pollen types (cumulative)	77.98	93.57	98.38	100		
PANN					13	0.002
MTWA					2	0.002
TANN					3.3	0.002
MTCO					1.5	0.002

#### 3.4.2 Establishment of the optimal model

The findings of this study, which are based on the WA-PLS method for model computation within an 800 km range, are presented in **Table 3**. According to the RMSEP and the coefficient of determination (R^2^), the optimal model for PANN, the optimal model is the second component (RESE = 62.903, R^2^ = 0.662, RMSEP = 67.276); for MTWA, the optimal model is the second component (RESE = 3.630, R^2^ = 0.511, RMSEP = 3.905); TANN is the second component (RESE = 2.975, R^2^ = 0.458, RMSEP = 3.190), while for MTCO, the optimal model is the second component (RESE = 2.581, R^2^ = 0.313, RMSEP = 2.766). An examination of the parameters reveals a linear relationship between observed and estimated values for four climate factors (**Figure 2c**), indicating their suitability for the quantitative reconstruction from pollen data of *Tamarix* cone sediment.

**Table 3:** The results of WA-PLS reconstruction.

Climate factors	Method	RESE	R^2^	RMSEP	Rand.t-test
PANN	Component 1	67.219	0.614	68.855	
	Component 2	62.903	0.662	67.276	0.001
	Component 3	60.201	0.690	70.947	0.606
	Component 4	59.141	0.701	73.806	0.747
	Component 5	59.011	0.702	76.034	0.967
MTWA	Component 1	3.883	0.442	3.974	
	Component 2	3.630	0.511	3.905	0.001
	Component 3	3.448	0.559	4.062	0.450
	Component 4	3.423	0.565	4.434	0.947
	Component 5	3.418	0.566	4.864	0.983
TANN	Component 1	3.170	0.386	3.249	
	Component 2	2.975	0.458	3.192	0.001
	Component 3	2.846	0.504	3.376	0.674
	Component 4	2.823	0.512	3.751	0.927
	Component 5	2.815	0.515	4.226	0.997
MTCO	Component 1	2.700	0.249	2.805	
	Component 2	2.581	0.313	2.766	0.002
	Component 3	2.513	0.348	2.872	0.691
	Component 4	2.485	0.362	3.086	0.882
	Component 5	2.477	0.367	3.370	0.978

#### 3.4.3 Matching and significance testing of surface soil pollen data with *Tamarix* cone sedimentary pollen data

In this study, Non-Metric Multi-Dimensional Scaling (NMDS) was employed to conduct matching and significance tests of surface soil pollen data with *Tamarix* cone sedimentary pollen, to access the congruence between surface soil pollen data and *Tamarix* cone sedimentary pollen data. The results indicate (**Figure 2d**) the selected topsoil pollen data encompasses the variability represented in the *Tamarix* cone sedimentary pollen; therefore the samples in this study can simulate the response model of pollen assemblages to past climatic changes and can be used for paleoclimate reconstruction in the study area. The significance test results show that (**Figure 2e**) the MTWA, TANN and MTCO reconstructed by the WA-PLS method passed the significance test, indicating that these ability to explain the changes in sediment pollen assemblages exceeds that of 95% of the stochastically structed variables, suggesting that the reconstruction results have a high degree of credibility. Therefore, in this study, we chose TANN, MTCO and MTWA for reconstruction. These results further confirm that surface soil pollen data can serve as a strong basis for the quantitative reconstruction of the paleoclimate in the southeastern Qaidam Basin.

#### 3.4.4 Quantitative reconstruction results

**Figure 7:**
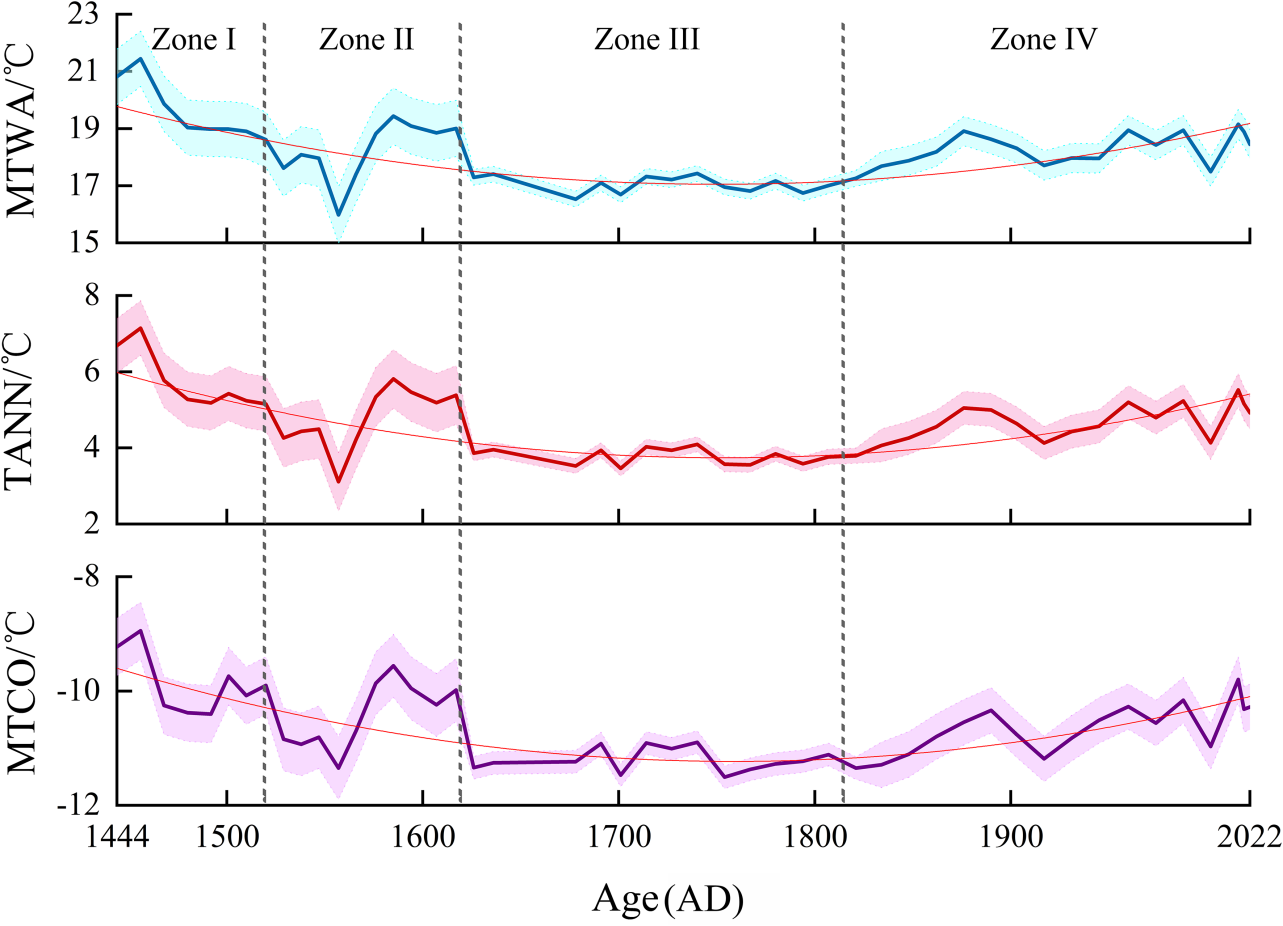
The temperature of the warmest month (MTWA), the mean annual temperature (TANN) and the temperature of the coldest month (MTCO) of *Tamarix* cone sediments in the Nuomuhong area derived from the Weighted Averaging Partial Least Squares (WA-PLS). The thick line is the test values, the thin line is a trend line, and the shading is the error range.

Based on the WA-PLS method, we quantitatively reconstructed the variation trend of MTWA, TANN and MTCO in the southeastern Qaidam Basin over the past 600 years. This record exhibits the temperature first decreases and then increases. The temperature record can be divided into four stages, as shown in **Figure 7** and described below.

Zone I (182–157 cm, 1444–1520 AD): The average MTWA was 19.6 °C (range: 18.6–21.4 °C), the average TANN was 5.7 °C (range: 5.2–7.1 °C), the average MTCO was -9.9 °C (range: -10.4–8.9 °C).

Zone II (157–126 cm, 1520–1617 AD): The average MTWA was 18.2 °C (range: 16.0–19.4 °C), the average TANN was 4.8 °C (range: 3.1–5.8 °C), the average MTCO was -10.4 °C (range: -11.3–9.6 °C).

Zone III (126–64 cm, 1617–1821 AD: The average MTWA was 17.1 °C (range: 16.5–17.4 °C), the average TANN was 3.8 °C (range: 3.5–4.1°C), the average MTCO was -11.2°C (range: -11.5– -10.9°C).

Zone IV (64–0 cm, 1821–2022 AD): The average MTWA was 18.3°C (range: 17.7–19.2°C), the average TANN was 4.7°C (range: 4.1–5.5°C), the average MTCO was -10.6°C (range: -11.3– -9.8°C).

## 4 Discussion

### 4.1 Influencing factors of environmental changes in the southeast Qaidam basin for the last 600 years

The Qaidam Basin is a large-scale continental depression basin, located in the northeastern margin of the Qinghai-Tibet Plateau, and it is one of the highest and driest deserts on Earth (Wang et al., 2013a). By analyzing the sedimentary pollen of *Tamarix* cones, the present study was conducted to reveal the climate change process in the Nuomuhong area of Qaidam Basin in the last 600 years.

**Figure 8:**
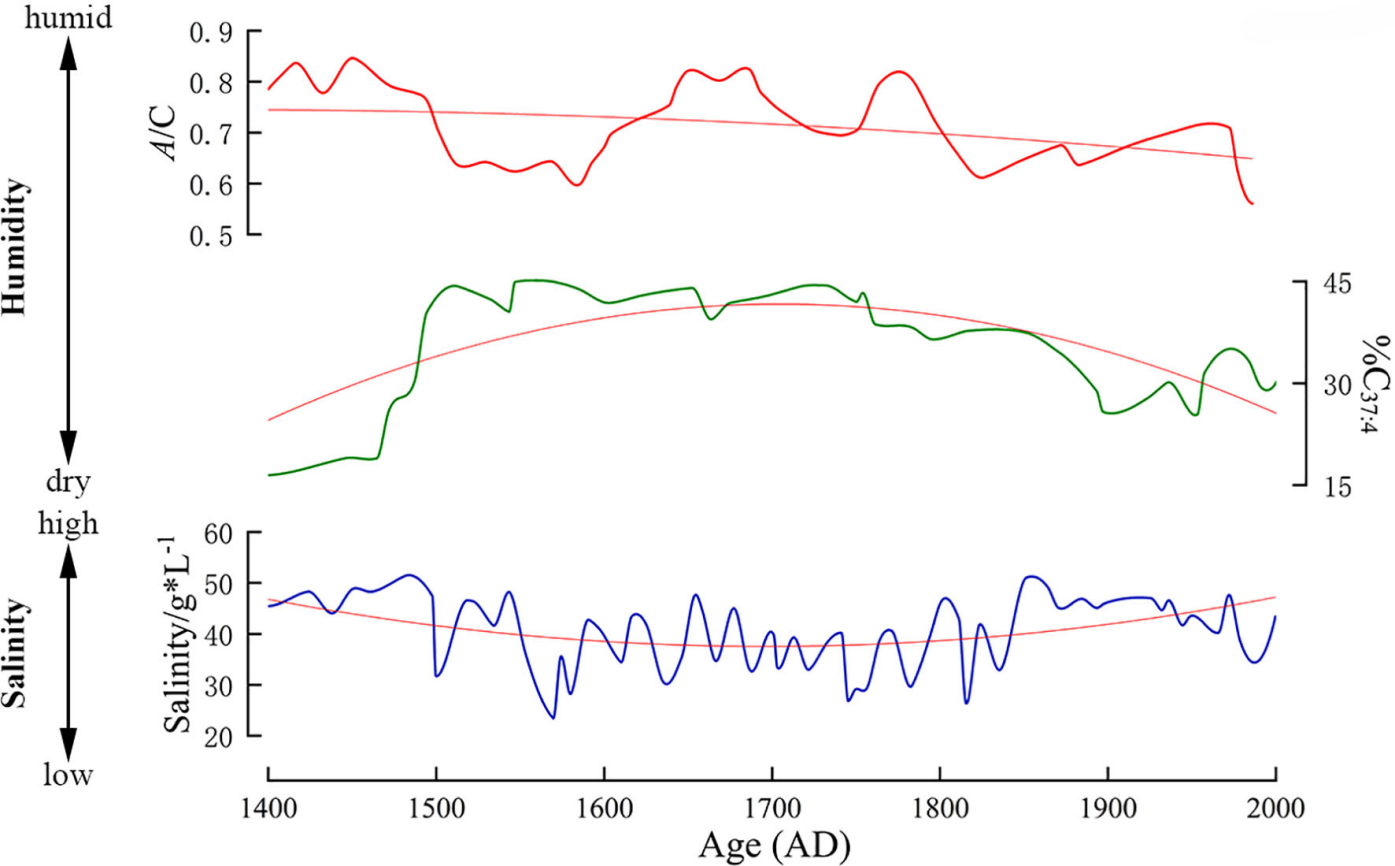
Coeval climate records from other regions of northwest China since the Little Ice Age, about the *Artemisia*/Chenopodiaceae (*A*/C) from the Hurleg Lake (Zhao, 2010), the alkenone-based %C_37:4_ records from Lake Sugan (He et al., 2013), and chironomid-based salinity reconstruction from Sugan Lake (Chen et al., 2019). The thick lines is the test values, the thin line is a trend line.

PCA revealed that since 1444 AD, environmental humidity has changed from a humid state to a dry state. The present results are consistent with the tree-ring δ^18^O series in the Qaidam Basin (Wang et al., 2013b), the precipitation series reconstructed by tree-ring width index (Shao et al., 2004), the effective humidity changes recorded by sediments in Sugan Lake (Chen et al., 2009) and others (**Figure 8**). The changes may be due to climate change in the Little Ice Age and atmospheric circulation. The temperature was lower during the early Little Ice Age, which increased the effective humidity by reducing evaporation. In the middle Little Ice Age, due to the warm intervals, the environmental humidity fluctuated slightly. For instance, the pollen content of *Artemisia*, which indicates a dry environment, first increased and then decreased. However, the overall environment remained humid during this period (Chen et al., 2008). From the late Little Ice Age to the present, a brief period of humid environment occurred due to a cold interval (Cui et al., 2018), and then gradually turned to drought. From 1444 to 2022 AD, soil salinity showed a trend of initial decrease followed by a subsequent increase. From 1444 to 1520 AD, the period was characterized by higher salinity; 1520–1617 AD, there were lower salinity; 1617–1821 AD, there were lower salinity; 1821–2022 AD, there were higher salinity. From 1444 to 1821 AD, the soil salinity showed a downward trend, which was mainly influenced by the cold and more humid climate during the Little Ice Age. During the Little Ice Age, the environment was more humid (Cheng et al., 2024), and the salinity decreased with the infiltration of water or runoff migration. This is consistent with coeval climate records from the chironomid-based salinity reconstruction from Sugan Lake (**Figure 8**). In general, soil salinity in arid areas is negatively correlated with environmental humidity (Leija and King, 2023); our results show that soil salinity has shown an increase trend since 1821 AD. This is because the Qaidam Basin is an arid area, with evaporation far exceeding precipitation. Therefore, apart from atmospheric precipitation, plant growth mainly relies on groundwater (Zhu et al., 2016). After the Little Ice Age, the temperature has risen, evaporation has increased, and plants have made more use of groundwater. Meanwhile, the salt in groundwater remains in the soil, and during the evaporation process, the salt in groundwater continuously migrates to the surface soil, resulting in an increase in soil salinity (Shokri-Kuehni et al., 2020). The WA-PLS method revealed that MTWA, TANN and MTCO showed an initial decrease followed by a subsequent increase trend (**Figure 7**). The MTWA, TANN and MTCO decreased by 5.9 °C, 3.7 °C and 2.6°C respectively from 1444 to 1821 AD, and increased by 2.6 °C, 2.1 °C and 1.7 °C respectively from 1821 to 2022 AD, which were in line with the trend of global warming. In general, the pollen assemblage of *Tamarix* cone sediments in the Nuomuhong area of the Qaidam Basin reflects the changes in humidity in this area over the past 600 years.

### 4.2 Significance of characteristic pollen ratio to environment

The ratios of *Artemisia*/Chenopodiaceae (*A*/C) and *Ephedra*/Chenopodiaceae (*Ep*/C) may reflect the degree of dryness and humidity in arid and semi-arid areas, and these ratios are often used as the basis for dividing the degree of drought. Chenopodiaceae is more tolerant of an arid environment than *Artemisia*. Lower *A*/C values indicate increased aridness. In general, *A*/C > 1 represents grassland areas, and *A*/C < 0.5 represents desert areas (Li et al., 2005). During the period from 1444 to 2022, the average pollen content of *Artemisia* and Chenopodiaceae was 39.68% (17–59.5%) in the Nuomuhong area, and the change of pollen *A*/C content indicated the local environment. However, the *A*/C ratio in the Nuomuhong area was higher (average value of 1.705, 0.289–6.194) because *Artemisia* pollen can be spread by wind dispersal (Liu et al., 2008), atmospheric circulation resulting in frequent windy weather in the study area and a large number of *Artemisia* pollen sediments. Thus, the *A*/C ratio in the Nuomuhong area had no environmental indicator significance. However, during the period from 1444 to 2022, the average content of *Ephedra* and Chenopodiaceae pollen was 23.71%, which was too low to be an environmental indicator.

As an indicator of soil salinity, we reconstructed the variation of soil salinity based on the content of *Nitraria* pollen. The result showed that from 1444 to 1520 AD, the content of *Nitraria* pollen was relatively high, indicating high soil salinity during this period. From 1520 to 1821 AD, the content of *Nitraria* pollen decreased, suggesting a decline in soil salt. From 1821 to 2022 AD, the content of *Nitraria* pollen increased, indicating that the soil salinity rose again during this period. *Nitraria* is an important desert shrub and one of the central ecological genera in the arid regions of northwest China. It has a high resistance to drought, salinity and wind erosion stresses (Chen et al., 2021). This is because the salt tolerance threshold of *Nitraria* is high, allowing it to grow in severely saline-alkali land (Yang et al., 2010). Its root system can directly absorb salt from the soil. When the soil salinity rises, *Nitraria* needs to increase the absorb Na^+^ to maintain its survival (Liu et al., 2014), resulting in relatively concentrated pollen production by *Nitraria* in salinized environments. In contrast, high soil salinity inhibits the growth of other plants, allowing the *Nitraria* to gain an advantage through salt tolerance. Zhao et al. (2020) demonstrated that *Nitraria* is dominant in saline-alkali areas and has strong representativeness. Therefore, *Nitraria* pollen can be used as a type of characteristic pollen to indicate the changes in soil salinity in arid areas.

### 4.3 The influencing of human activities in the Southeast Qaidam basin for the last 600 years

The Qinghai-Tibet Plateau where the Qaidam Basin is located, as one of the regions with the lowest population density in the world, has a natural environment that greatly restricts large-scale settlement of people (Hou, 2016). Lake sediments in the Angren Basin indicate that the cold and dry climate of the Little Ice Age inhibited the development of agriculture and crop cultivation, and the human population on the Qinghai-Tibet Plateau was very small between 1480 and 1820 AD (Li et al., 2024). According to the research of Yang et al. (2025), our sampling site is located in the exclusion area (nature reserve) of the potential area for human activities and the moderately suitable and marginally suitable areas of the suitability level for human activities. Meanwhile, they are confronted with the problem of resource-based water shortage. These areas are only suitable for the development of low-water-consuming industrial and mining activities and are difficult to support large-scale population agglomeration. The potential for further expanding human activities is relatively small. However, with the development of productivity in the middle and late Qing Dynasty (Jin et al., 2022) and the temperature increasing which was relatively suitable for human life, the traces of human activities relatively increased after the end of the Little Ice Age (Li et al., 2024), which in turn affected vegetation (Wende et al., 2023). Our pollen assemblage result showed that the pollen quantity of Rosaceae increased around 2000AD (**Figure 3**), which is related to the fruit tree planting in the oases around the sampling sites during the corresponding period (Guo et al., 2011). However, since this study passed the matching and significance tests of surface soil pollen data, the impact of human activities on vegetation does not affect the climate reconstruction results of this study.

## 5 Conclusions

This study reconstructs the environmental changes in the southeastern Qaidam Basin using sedimentary veins and the pollen assemblage in *Tamarix* cone sediments over the past 600 years. The conclusions are as follows:

1. Based on the ^210^Pb dating, ^14^C dating and nuclear events, the age of the Nuomuhong *Tamari*x cone profile was determined to be 1444–2022 AD.
2. According to the cluster analysis and PCA combined with WA-PLS, the environmental change in the Nuomuhong area from 1444 to 2022 AD was divided into four stages. From 1444 to 1520 AD, with higher salinity and humid conditions (MTWA of 18.6–21.4°C, TANN of 5.2–7.1°C, and MTCO of -10.4–8.9°C). From 1520 to 1617 AD, with lower salinity and a humid conditions (MTWA of 16.0–19.40°C, TANN of 3.102–5.80°C, MTCO of -11.30–9.6°C). From 1617 to 1821 AD, with progressive aridification with reduced salinity (MTWA of 16.5–17.4°C, TANN of 3.5–4.1°C, MTCO of -11.5–10.9°C). And from 1821 to 2022 AD, with increasing salinity and a drier conditions (MTWA of 17.7–19.2°C, TANN of 4.1–5.5°C, MTCO of -11.3–9.8°C. These results suggested that the environmental changes may be related to climate and groundwater changes mainly caused by the Little Ice Age.
3. The characteristic pollen ratio is influenced by pollen content and pollen source. Due to the relatively lower content of *Ephedra* in the Nuomuhong area and the atmospheric circulation transporting a large amount of *Artemisia* pollen over a long distance, the *Ep/*C and *A*/C ratios lack environmental indication significance in the Nuomuhong area. And the *Nitraria* pollen can be used as characteristic pollen to indicate the changes in soil salinity in arid areas.

This study provides a unique perspective for environmental change in Qaidam Basin, although our method has limitations due to incomplete sedimentary profile and insufficiently precise pollen identification. In our future work, we will try to conduct cross-regional comparisons of the sedimentary environment of *Tamarix* cones and analyze the patterns of environmental change in arid regions.

## Data Availability

The datasets presented in this study can be found in online repositories. The names of the repository/repositories and accession number(s) can be found in the article/supplementary material.
